# Supplementary material for: YeeE-like bacterial SoxT proteins mediate sulfur import for oxidation and signal transduction
Source: Commun Biol. 2024 Nov 21;7:1548. doi: 10.1038/s42003-024-07270-7 (PMC11582611; doi:10.1038/s42003-024-07270-7)
Supplement: Supplementary file 2 — Supplementary Information [file 42003_2024_7270_MOESM2_ESM.pdf]

YeeE-like bacterial SoxT proteins mediate sulfur import for oxidation and signal transduction

Jingjing Li, Fabienne Göbel, Hsun Yun Hsu, Julian Nikolaus Koch, Natalie Hager, Wanda Antonia Flegler, Tomohisa Sebastian Tanabe, Christiane Dahl*

Corresponding author: ChDahl@uni-bonn.de

Institut für Mikrobiologie & Biotechnologie, Rheinische Friedrich-Wilhelms-Universität Bonn, Bonn, Germany

Supplementary Figures and Tables:

Supplementary Fig. 1: Alignment of YeeE/YedE family proteins.

Supplementary Fig. 2: Volcano plot of differentially expressed genes for the *H. denitrificans* Δ*tsdA* reference strain in the absence versus the presence of thiosulfate.

Supplementary Fig. 3. Transcript abundance changes of genes encoding transcriptional regulators and neighboring genes from *Hyphomicrobium denitrificans* Δ*tsdA*

Supplementary Fig. 4. Comparison of *Hyphomicrobium denitrificans* YeiH (Hden_0834, UniProt D8JU61) with SuyZ from *Paracoccus pantotrophus* NKNCYSA (beige, GenBank accession AY704413).

Supplementary Fig. 5: Transcript abundance changes of genes encoding PmpA and PmpB and neighboring genes from *Hyphomicrobium denitrificans* Δ*tsdA*.

Supplementary Fig. 6: Growth of *H. denitrificans* reference and mutant strains on methanol.

Supplementary Fig. 7: Growth and thiosulfate consumption of *H. denitrificans* reference and mutant strains lacking *soxT1B*.

Supplementary Fig. 8: Growth and thiosulfate consumption of *H. denitrificans* reference and mutant strains lacking *soxT1A*.

Supplementary Fig. 9 Overlay of YeeE-like protein structures.

Supplementary Table 1: mRNAseq analysis of *H. denitrificans ΔtsdA,* part 1.

Supplementary Table 2: mRNAseq analysis of *H. denitrificans ΔtsdA,* part 2.

Supplementary Table 3: Strains, primers and plasmids

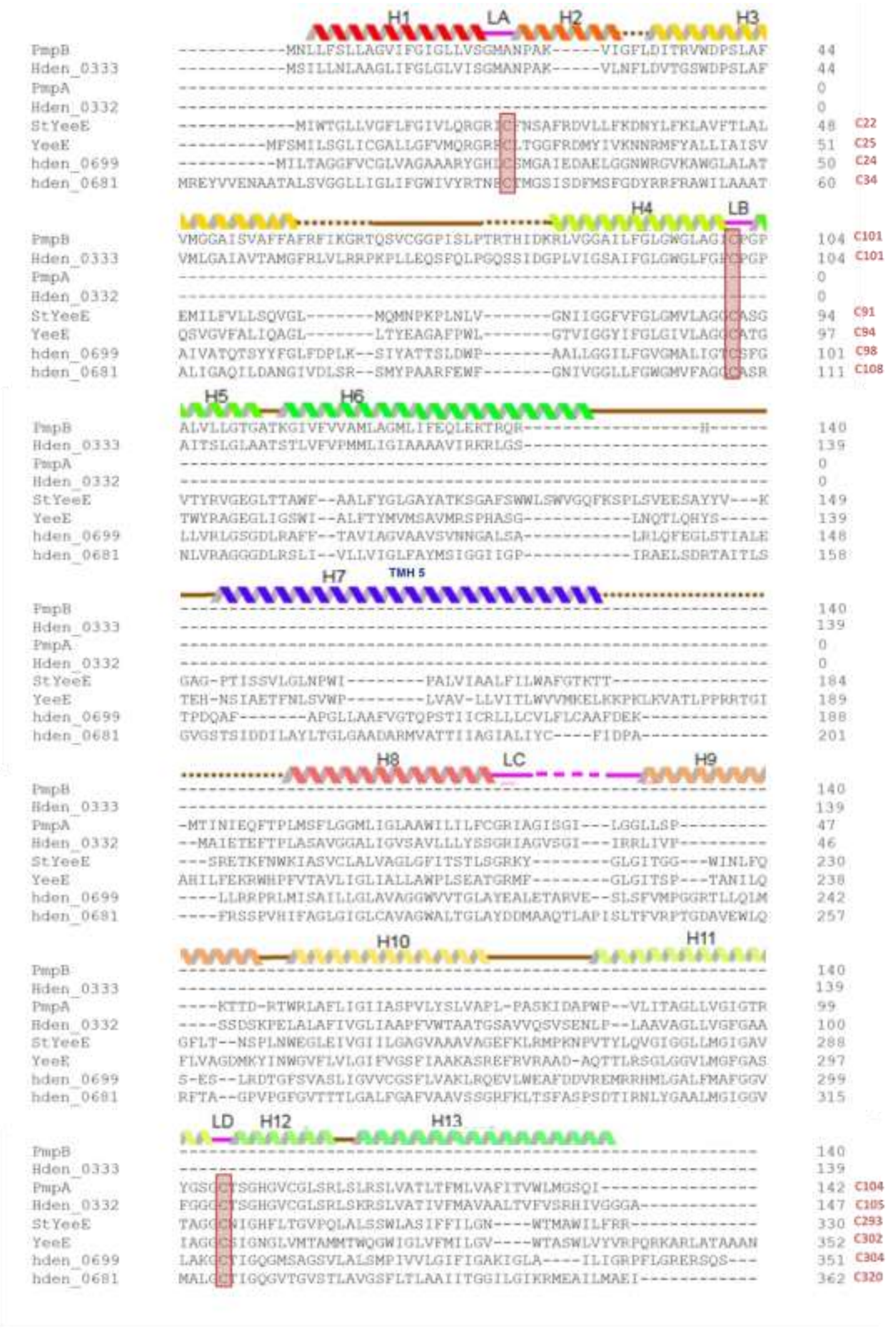

Supplementary Fig. 1. Alignment of YeeE/YedE family proteins. Amino acid sequences of PmpA and PmpB from *Serratia* sp. (Ser39006_020715 and Ser39006_02071520) and *Hyphomicrobium denitrificans* (Hden_0332 and Hden_0333), SoxT1A (Hden_0681) and SoxT1B (Hden_0699) from *H. denitrificans*, YeeE (TsuA, b2013) from *E. coli* K12 and StYeeE (Spith_0734) from *Spirochaeta thermophila* DSM 6578 were aligned with Clustal Omega (EMBL-EBI). Conserved cysteines are marked in red. The secondary structure of StYeeE with loops LA to LD and α-helices H1 to H13 was taken from Tanaka *et al.,* 2020 [1]. Helices 1, 3, 4, 6,7, 8, 10, 11 and 13 are membrane-spanning.

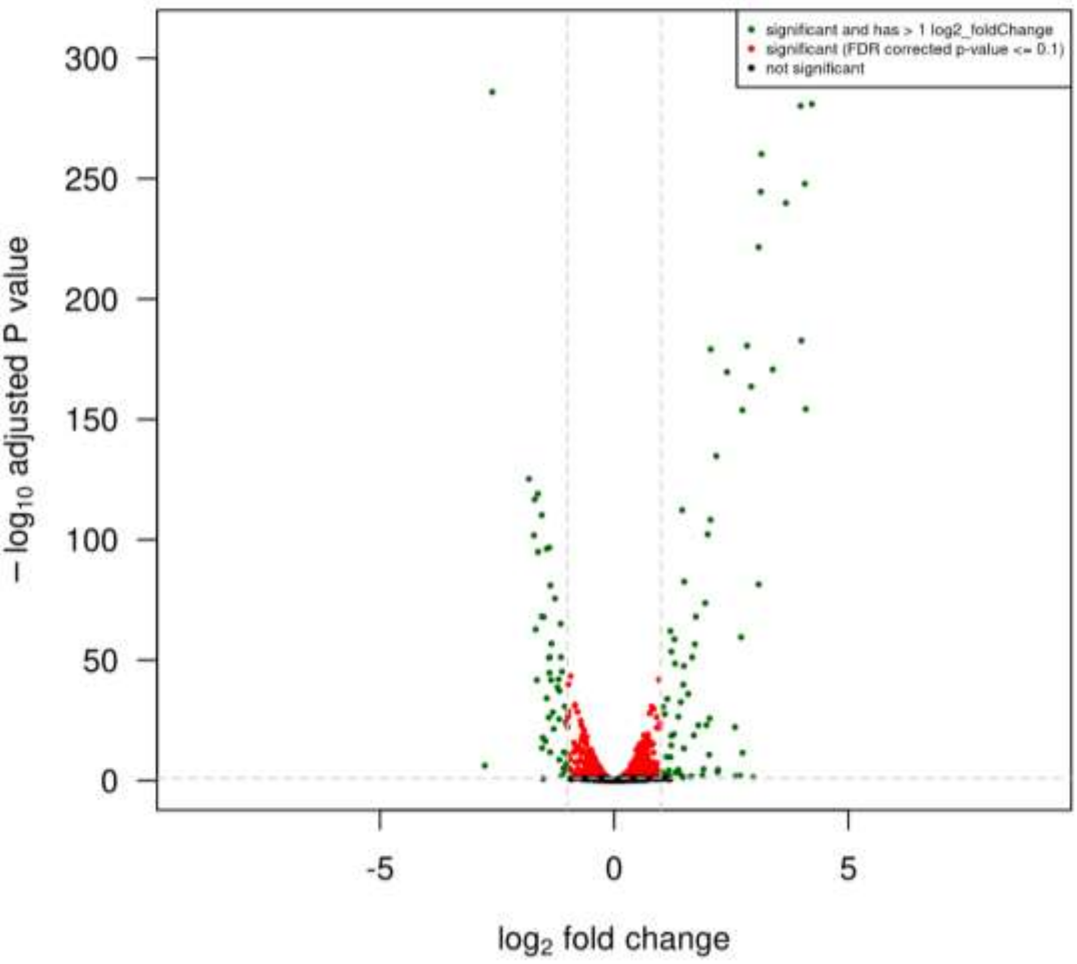

Supplementary Fig. 2. Volcano plot of differentially expressed genes for the *H. denitrificans* Δ*tsdA* reference strain in the absence versus the presence of thiosulfate. log2-fold change threshold =1, Benjamini-Hochberg corrected p-value = 0.1.

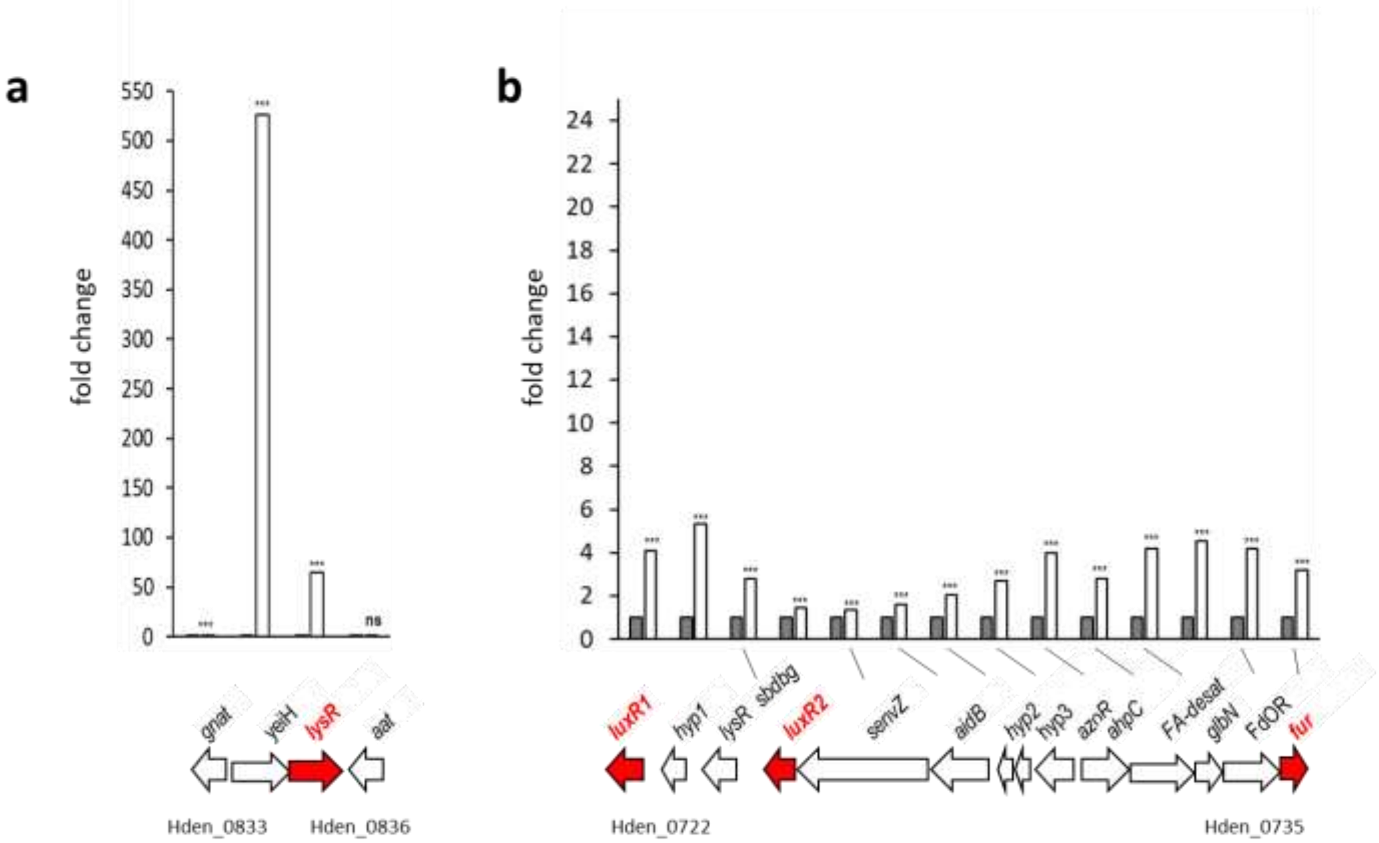

Supplementary Fig. 3. Transcript abundance changes of genes encoding transcriptional regulators and neighboring genes from *Hyphomicrobium denitrificans* ΔtsdA. Columns in gray show the reference value for cells grown in the absence of thiosulfate, white columns apply to cells grown with 2 mM thiosulfate. The experiment was conducted in duplicate (n=2), each time using mRNA preparations from two different cultures. Adjusted *p* values for statistically significant changes were all below 0.001 (Supplementary Table 2) and are indicated by three asterisks (***p < 0.001); ns, not significant. **a,** *gnat*, acyl-CoA N-acetyltransferase domain; *yeiH,* proposed sulfate/sulfite exporter; *lysR*, transcriptional regulator LysR family; the majority of these proteins appear to be transcription activators and most are known to negatively regulate their own expression; *aat*, leucyl/phenylalanyl-t-RNA/protein transferase. **b,** *luxR1*, LuxR family transcriptional regulator; most luxR-type regulators act as transcription activators, but some can be repressors or have a dual role for different sites; *hyp1*, hypothetical periplasmic protein; *lysR sbdg*, substrate binding domain LysR family of prokaryotic transcriptional regulatory proteins; *luxR2*, LuxR family response regulator; *envZ*, multi-sensor signal transduction histidine kinase; *aidB,* acyl-CoA dehydrogenase domain related to the alkylation response protein AidB; *hyp2* and *hyp3*, hypothetical proteins; *azoR*, FMN-dependent NADH:quinone oxidoreductase; *ahpC*, peroxiredoxin, alkyl hydroperoxide reductase subunit C; *FA-desat*, fatty acid desaturase; *glbN*, globin, truncated bacterial like; *FdOR*, 2Fe-2S ferredoxin domain containing FAD/NAD(P)-binding oxidoreductase; *fur*, ferric uptake regulator, Fur family, iron-responsive DNA-binding repressor protein;

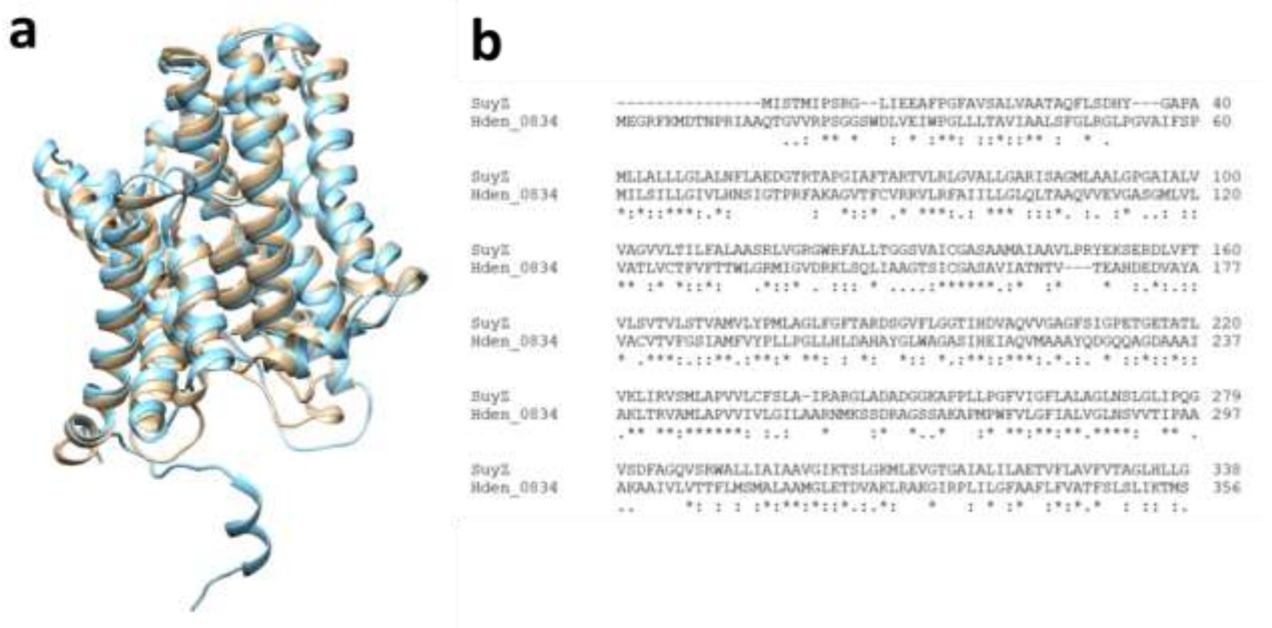

Supplementary Fig. 4. Comparison of *Hyphomicrobium denitrificans* YeiH (Hden_0834, UniProt D8JU61) with SuyZ from *Paracoccus pantotrophus* NKNCYSA (beige, GenBank accession AY704413). a. Alphafold[2] structures of HdYeiH (light blue) and PpSuyZ (beige). Structures were overlayed and visua-lized by Chimera 1.14[3]. **b.** Sequence alignment of SuyZ and HdYeiH (Hden_0834) created by Clustal Omega[4].

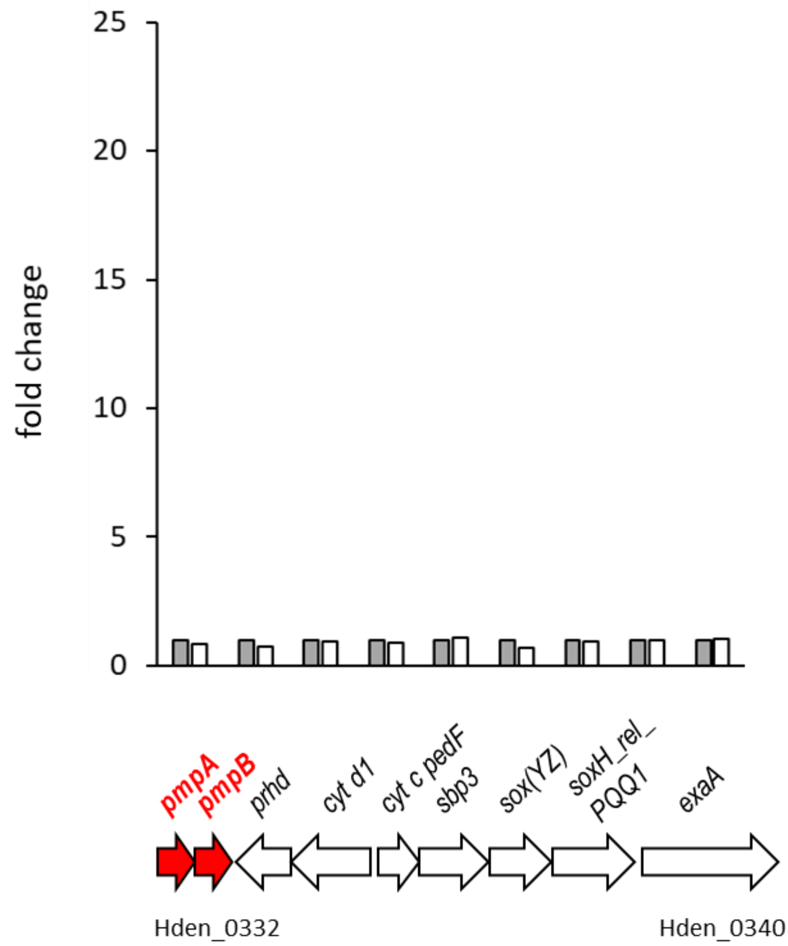

Supplementary Fig. 5. Transcript abundance changes of genes encoding PmpA and PmpB and neighboring genes from *Hyphomicrobium denitrificans* Δ*tsdA*. Columns in gray show the reference value for cells grown in the absence of thiosulfate, white columns apply to cells grown with 2 mM thiosulfate. The experiment was conducted in duplicate, each time using mRNA preparations from two different cultures. Adjusted *p* values were all above 0.05, indicating no significant changes. *prhd*, periplasmic rhodanese-like domain-containing sulfurtransferase with a CXXXCW motif, consistent with a possible role in redox cofactor binding (IPR001763 and IPR022376); *cyt d1*, periplasmic protein with a cytochrome *d1* heme domain and a YVTN beta-propeller repeat (TIGR03866); *pedF*, cytochrome c_{550} (TIGR04494), periplasmic electron carrier; *sbp3*, extracellular substrate-binding protein family 3 (IPR001638). *sox(YZ),* Sox(YZ) fusion protein (TIGR04557); *soxH_rel_PQQ1*, encodes a potential Zn metallohydrolase of the same family as the SoxH protein (TIGR04558) associated with thiosulfate oxidation[5]. *prhd*, *cyt d1*, *cyt c550*, *sox(YZ)*, and *soxH_rel_PQQ1* show relationships by phylogenetic profiling and conserved gene neighborhoods with transport systems for alcohols metabolized by PQQ-dependent enzymes (here probably the enzyme encoded by *exaA*), that have a Cys-Cys motif (TIGR03075) for electron transfer to c_{550} family cytochromes.

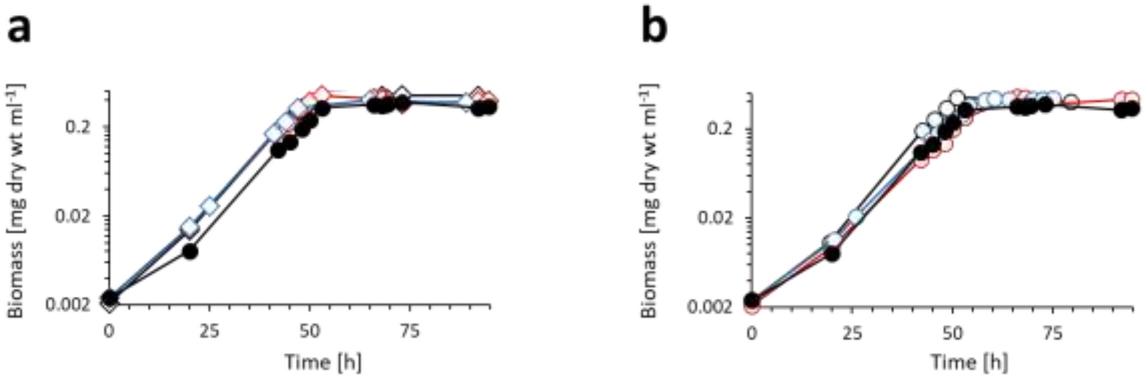

Supplementary Fig. 6. Growth of *H. denitrificans* reference and mutant strains on methanol. a. Growth curves are compared for the reference strain *H. denitrificans* Δ*tsdA* (black filled circles) and strains lacking gene *soxT1B*: *H. denitrificans* Δ*tsdA* Δ*soxT1B* (black open diamonds), *H. denitrificans* Δ*tsdA* Δ*soxT1B* Δ*soxR* (blue open diamonds) and *H. denitrificans* Δ*tsdA* Δ*soxT1B* Δ*shdrR* (red open diamonds). **b.** Growth curves are compared for the reference strain *H. denitrificans* Δ*tsdA* (black filled circles) and strains lacking gene *soxT1A*: *H. denitrificans* Δ*tsdA* Δ*soxT1A* (black open circles), *H. denitrificans* Δ*tsdA* Δ*soxT1A* Δ*soxR* (blue open circles) and *H. denitrificans* Δ*tsdA* Δ*soxT1A* Δ*shdrR* (red open circles). Error bars indicating SD for three replicates are too small to be visible for the determination of biomass.

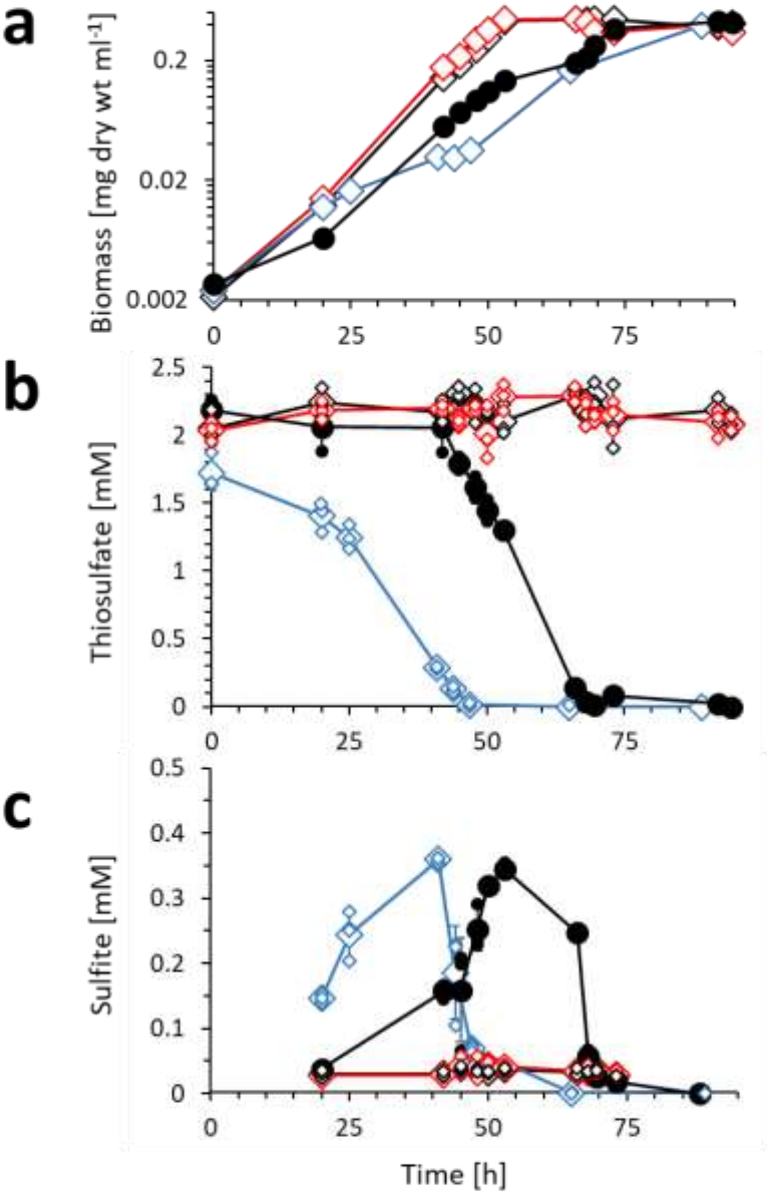

Supplementary Fig. 7. Growth and thiosulfate consumption of *H. denitrificans* reference and mutant strains lacking *soxT1B*. a. Growth curves on medium containing 2 mM thiosulfate. Error bars indicating SD for three replicates are too small to be visible for the determination of biomass. **b.** Thiosulfate consumption **c**. Sulfite production. Symbols identifying strains: *H. denitrificans* Δ*tsdA* (black filled circles), *H. denitrificans* Δ*tsdA* Δ*soxT1B* (black open diamonds), *H. denitrificans* Δ*tsdA* Δ*soxT1B* Δ*soxR* (blue open diamonds) and *H. denitrificans* Δ*tsdA* Δ*soxT1B* Δ*shdrR* (red open diamonds). In **b** and **c**, data was measured using $n = 3$ experiments and is presented with the individual measurements (small symbols) and as the mean value of these measurements ± SD (big symbols). Precultures contained 2 mM thiosulfate.

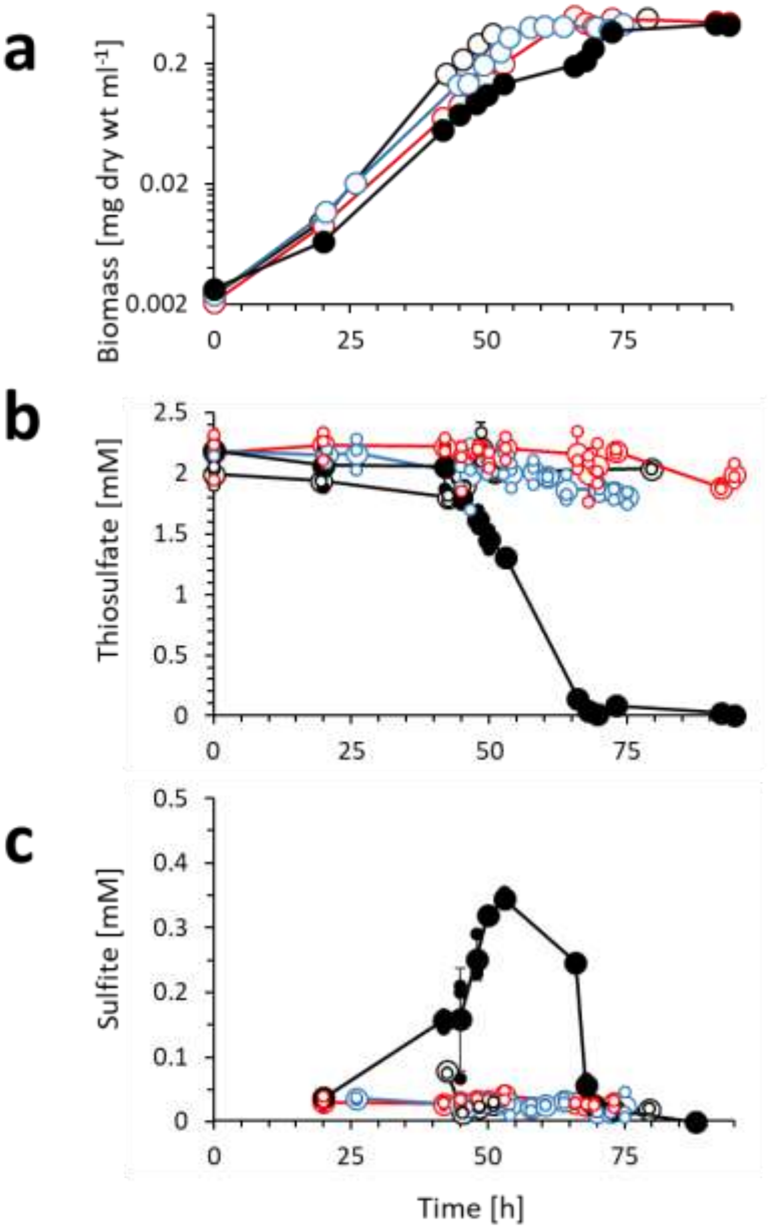

Supplementary Fig. 8. Growth and thiosulfate consumption of *H. denitrificans* reference and mutant strains lacking *soxT1A*. a. Growth curves on medium containing 2 mM thiosulfate. Error bars indicating SD for three replicates are too small to be visible for the determination of biomass. **b.** Thiosulfate consumption **c**. Sulfite production. Symbols identifying strains: *H. denitrificans* Δ*tsdA* (black filled circles), *H. denitrificans* Δ*tsdA* Δ*soxT1A* (black open circles), *H. denitrificans* Δ*tsdA* Δ*soxT1A* Δ*soxR* (blue open circles) and *H. denitrificans* Δ*tsdA* Δ*soxT1B* Δ*shdrR* (red open circles). In **b** and **c**, data was measured using *n* = 3 experiments and is presented with the individual measurements (small symbols) and as the mean value of these measurements ± SD (big symbols). Precultures contained 2 mM thiosulfate.

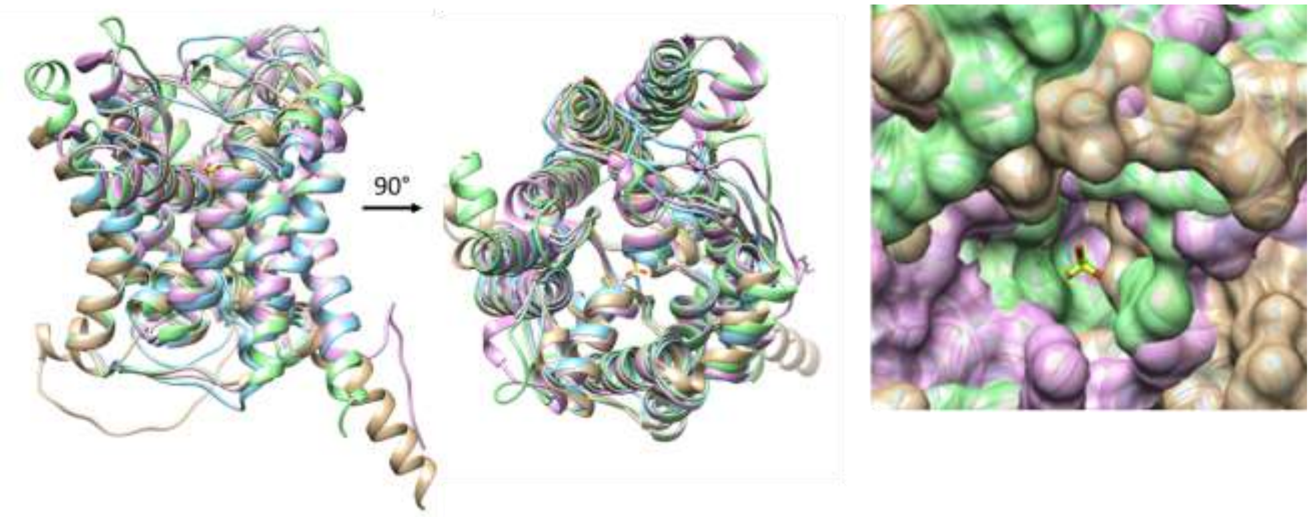

Supplementary Fig. 9. Overlay of YeeE-like protein structures. The crystal structure of *Spirochaeta thermophila* TsuA (YeeE) with thiosulfate bound (6LEO[1], light blue) was matched with the structures for *E. coli* YeeE (beige), *H. denitrificans* SoxT1A (green) and *H. denitrificans* SoxT1B (violet) predicted by Alphafold[2]. The image on the right displays the protein surfaces with 30% transparency and reveals free access for thiosulfate and/or other potential substrates to the (proposed) binding site for all cases. Structures were matched and visualized by Chimera 1.14[3].

Supplementary Table 1. mRNAseq analysis of *H. denitrificans ΔtsdA*, part 1. Genes with lower mRNA abundances in thiosulfate containing medium in comparison to thiosulfate free medium.

Locus tag NCBI	Annotation	Fold change	log_2_fold change	p_value	adj_p_value
Hden_0086	group II truncated hemoglobin	0.34	-1.54	1.36E-15	3.34E-14
Hden_0095	HPF/RaiA family ribosome-associated protein	0.31	-1.68	2.70E-65	1.79E-63
Hden_0096	zinc-dependent alcohol dehydrogenase family protein	0.31	-1.71	1.75E-104	1.56E-102
Hden_0097	flavin reductase family protein	0.32	-1.65	3.84E-44	1.85E-42
Hden_0099	PAS domain-containing protein	0.47	-1.07	7.24E-14	1.60E-12
Hden_0138	hypothetical protein	0.46	-1.12	2.13E-03	9.53E-03
Hden_0432	hypothetical protein	0.35	-1.51	7.50E-02	1.72E-01
Hden_0554	beta-ketoacyl-ACP synthase	0.39	-1.37	8.25E-14	1.81E-12
Hden_0555	beta-ketoacyl-ACP synthase	0.38	-1.39	1.84E-28	6.70E-27
Hden_0556	zinc-binding dehydrogenase	0.37	-1.44	1.58E-36	7.02E-35
Hden_0557	beta-ketoacyl-ACP synthase	0.44	-1.17	9.49E-28	3.38E-26
Hden_0558	beta-ketoacyl-ACP synthase	0.38	-1.39	2.03E-53	1.12E-51
Hden_0559	beta-hydroxyacyl-ACP dehydratase	0.34	-1.54	1.21E-70	8.68E-69
Hden_0560	acyl carrier protein	0.31	-1.70	1.88E-119	1.92E-117
Hden_0561	SDR family oxidoreductase	0.28	-1.82	4.85E-128	5.29E-126
Hden_0562	HAD-IIIC family phosphatase	0.39	-1.36	1.22E-83	9.35E-82
Hden_0567	U32 family peptidase	0.49	-1.02	5.71E-09	8.10E-08
Hden_0568	U32 family peptidase	0.47	-1.08	9.51E-07	9.79E-06
Hden_0570	cyclic nucleotide-binding domain-containing protein	0.39	-1.35	3.58E-44	1.76E-42
Hden_0572	hypothetical protein	0.35	-1.53	5.98E-20	1.73E-18
Hden_0573	4Fe-4S binding protein	0.49	-1.03	2.07E-26	7.21E-25
Hden_0574	cupredoxin domain-containing protein	0.38	-1.38	3.70E-47	1.92E-45
Hden_0575	iron transporter	0.37	-1.44	6.51E-99	5.50E-97
Hden_0576	oxygen-independent coproporphyrinogen III oxidase	0.32	-1.62	1.38E-97	1.13E-95
Hden_0581	cytochrome c	0.36	-1.47	1.69E-18	4.68E-17
Hden_0589	hypothetical protein	0.44	-1.17	1.49E-10	2.47E-09
Hden_0590	NnrS family protein	0.50	-1.01	1.98E-05	1.55E-04
Hden_0591	copper-containing nitrite reductase	0.39	-1.37	1.02E-53	5.92E-52
Hden_0592	host attachment family protein	0.40	-1.31	1.26E-30	4.91E-29
Hden_0595	helix-turn-helix domain-containing protein	0.38	-1.38	2.00E-99	1.73E-97
Hden_0925	MFS transporter	0.40	-1.34	2.23E-59	1.37E-57
Hden_0976	gamma-glutamyl-gamma-aminobutyrate hydrolase family protein	0.44	-1.17	1.44E-39	6.59E-38
Hden_1046	GTP-binding protein	0.16	-2.60	4.68E-289	1.13E-286
Hden_1047	sulfate adenylyltransferase subunit CysD	0.35	-1.51	1.81E-70	1.25E-68
Hden_1055	hypothetical protein	0.44	-1.19	2.16E-44	1.09E-42
Hden_1491	NADPH-dependent assimilatory sulfite reductase hemoprotein subunit	0.32	-1.63	7.95E-122	8.40E-120
Hden_1773	radical SAM protein	0.50	-1.01	5.37E-13	1.10E-11
Hden_1841	universal stress protein	0.48	-1.06	4.24E-33	1.77E-31
Hden_2047	cytochrome-c oxidase 2C cbb3-type subunit III	0.46	-1.14	1.05E-53	6.03E-52
Hden_2048	cbb3-type cytochrome c oxidase subunit 3	0.43	-1.21	4.63E-41	2.14E-39
Hden_2049	cytochrome-c oxidase 2C cbb3-type subunit II	0.45	-1.14	1.21E-67	8.15E-66
Hden_2050	cytochrome-c oxidase%2C cbb3-type subunit I	0.42	-1.26	3.47E-78	2.61E-76
Hden_2136	DHA2 family efflux MFS transporter permease subunit	0.41	-1.29	1.26E-23	3.97E-22
Hden_2177	Crp/Fnr family transcriptional regulator	0.46	-1.12	1.21E-47	6.37E-46
Hden_2272	membrane protein	0.49	-1.04	9.08E-06	7.75E-05
Hden_2394	lysozyme inhibitor LprI family protein	0.15	-2.76	5.63E-08	7.02E-07
Hden_2827	ferric reductase-like transmembrane domain-containing protein	0.34	-1.55	5.87E-113	5.66E-111

Supplementary Table 2. mRNAseq analysis of *H. denitrificans ΔtsdA*, part 2. Genes with higher mRNA abundances in thiosulfate containing medium in comparison to thiosulfate free medium

Locus tag NCBI	Annotation	Fold change	log_2_fold change	p_value	adj_p_value
Hden_0441	glycosyltransferase	6.04	2.59	4.91E-03	1.91E-02
Hden_0444	glycosyltransferase family 4 protein	2.09	1.07	6.44E-03	2.40E-02
Hden_0457	hypothetical protein	4.65	2.22	3.21E-06	2.99E-05
Hden_0460	DUF983 domain-containing protein	2.42	1.27	1.97E-21	6.04E-20
Hden_0523	zf-HC2 domain-containing protein	2.61	1.38	7.21E-05	4.99E-04
Hden_0525	catalase family peroxidase	2.24	1.17	8.70E-06	7.46E-05
Hden_0678	hypothetical protein	6.69	2.74	1.58E-13	3.38E-12
Hden_0679	DsbA family protein	8.49	3.09	4.28E-84	3.36E-82
Hden_0680	sulfur transferase domain-containing protein	12.69	3.67	8.60E-243	1.45E-240
Hden_0681	YeeE/YedE family protein	18.94	4.24	0.00E+00	0.00E+00
Hden_0683	radical SAM protein LipS1	16.81	4.07	7.46E-251	1.40E-248
Hden_0684	NAD(P)/FAD-dependent oxidoreductase LipT	17.04	4.09	4.28E-157	5.17E-155
Hden_0685	radical SAM protein LipS2	15.85	3.99	0.00E+00	0.00E+00
Hden_0686	lipoate--protein ligase family protein sLpl(AB)	16.65	4.06	7.66E-319	1.99E-316
Hden_0687	GMP synthase - glutamine amidotransferase domain-like protein LipX	15.98	4.00	1.23E-185	1.89E-183
Hden_0689	sHdrC1	16.09	4.01	0.00E+00	0.00E+00
Hden_0690	sHdrB	17.82	4.16	0.00E+00	0.00E+00
Hden_0691	sHdrA	19.29	4.27	0.00E+00	0.00E+00
Hden_0692	sHdrH	18.64	4.22	5.23E-284	1.18E-281
Hden_0693	sHdrC2	15.79	3.98	2.81E-283	5.93E-281
Hden_0694	sHdrB2	13.34	3.74	0.00E+00	0.00E+00
Hden_0695	sHdrI	10.45	3.39	1.48E-173	2.00E-171
Hden_0696	LbpA2	8.49	3.09	1.81E-224	2.91E-222
Hden_0697	cytochrome P450	7.14	2.84	1.62E-183	2.38E-181
Hden_0698	sulfurtransferase TusA family protein	7.60	2.93	1.86E-166	2.32E-164
Hden_0701	SoxS	6.54	2.71	4.74E-62	3.02E-60
Hden_0702	sulfur oxidation c-type cytochrome SoxX	6.68	2.74	1.21E-156	1.41E-154
Hden_0703	sulfur oxidation c-type cytochrome SoxA	8.85	3.15	3.34E-263	6.63E-261
Hden_0704	thiosulfate oxidation carrier protein SoxY	8.97	3.16	0.00E+00	0.00E+00
Hden_0705	thiosulfate oxidation carrier complex protein SoxZ	8.76	3.13	1.60E-247	2.84E-245
Hden_0706	thiosulfohydrolase SoxB	7.29	2.86	0.00E+00	0.00E+00
Hden_0719	TIGR01244 family sulfur transferase	2.79	1.48	3.31E-42	1.55E-40
Hden_0720	sulfite exporter TauE/SafE family protein	2.59	1.37	9.37E-29	3.52E-27
Hden_0721	MBL fold metallo-hydrolase	2.99	1.58	2.59E-38	1.16E-36
Hden_0722	response regulator transcription factor	4.08	2.03	1.03E-12	2.04E-11
Hden_0723	hypothetical protein	5.31	2.41	1.61E-172	2.09E-170
Hden_0724	substrate-binding domain-containing protein	2.80	1.49	2.09E-15	5.03E-14
Hden_0728	hypothetical protein	2.06	1.04	6.78E-33	2.76E-31
Hden_0729	hypothetical protein	2.68	1.42	6.37E-35	2.76E-33
Hden_0730	NAD(P)H-dependent oxidoreductase	3.99	2.00	6.42E-105	5.86E-103
Hden_0731	peroxiredoxin	2.82	1.49	3.20E-85	2.58E-83
Hden_0732	fatty acid desaturase	4.18	2.06	5.43E-182	7.65E-180
Hden_0733	group 1 truncated hemoglobin	4.53	2.18	1.53E-137	1.72E-135
Hden_0734	2Fe-2S iron-sulfur cluster binding domain-containing protein	4.17	2.06	5.70E-111	5.35E-109
Hden_0735	transcriptional repressor	3.17	1.67	1.19E-53	6.72E-52
Hden_0737	alpha/beta fold hydrolase	3.30	1.72	3.97E-59	2.40E-57
Hden_0738	hypothetical protein	5.98	2.58	2.10E-24	6.83E-23
Hden_0739	flavin reductase family protein	3.25	1.70	5.67E-21	1.69E-19
Hden_0742	SCO family protein	3.85	1.95	2.72E-76	2.00E-74
Hden_0743	selenium-binding protein	7.36	2.88	0.00E+00	0.00E+00
Hden_0748	cell envelope integrity protein TolA	2.03	1.02	3.91E-05	2.85E-04
Hden_0783	PQQ-binding-like beta-propeller repeat protein	2.46	1.30	4.87E-51	2.65E-49
Hden_0784	thiamine pyrophosphate-dependent dehydrogenase E1 component subunit alpha	2.81	1.49	5.40E-50	2.90E-48
Hden_0785	alpha-ketoacid dehydrogenase subunit beta	2.44	1.29	3.49E-61	2.19E-59
Hden_0786	acetoin dehydrogenase dihydrolipoyllysine-residue acetyltransferase subunit	2.33	1.22	4.78E-56	2.84E-54
Hden_0788	thiazole synthase	2.59	1.37	3.40E-06	3.13E-05
Hden_0791	dihydrolipoyl dehydrogenase	2.33	1.22	9.79E-21	2.88E-19
Hden_0792	hypothetical protein	2.30	1.20	1.29E-64	8.40E-63
Hden_0796	methanol/ethanol family PQQ-dependent dehydrogenase	2.12	1.08	6.90E-30	2.62E-28
Hden_0834	YeiH family protein	525.97	9.04	0.00E+00	0.00E+00
Hden_0835	LysR family transcriptional regulator	65.57	6.03	0.00E+00	0.00E+00
Hden_0914	hypothetical protein	6.46	2.69	2.83E-03	1.20E-02

Hden_1114	hypothetical protein	3.08	1.62	1.14E-02	3.84E-02
Hden_1133	Do family serine endopeptidase	2.74	1.45	4.60E-115	4.58E-113
Hden_1509	metal-sensitive transcriptional regulator	2.16	1.11	6.08E-02	1.47E-01
Hden_1967	hypothetical protein	2.72	1.45	3.95E-03	1.59E-02
Hden_2058	isocitrate lyase	2.24	1.17	2.35E-02	6.90E-02
Hden_2164	AraC family transcriptional regulator	2.01	1.01	3.10E-02	8.60E-02
Hden_2373	DUF4118 domain-containing protein	2.26	1.18	3.47E-03	1.42E-02
Hden_2458	hypothetical protein	7.83	2.97	1.25E-02	4.16E-02
Hden_2475	PsiF family protein	3.92	1.97	2.80E-25	9.57E-24
Hden_2542	class I SAM-dependent methyltransferase	4.62	2.21	4.34E-05	3.14E-04
Hden_2565	Spy/CpxP family protein refolding chaperone	2.19	1.13	3.46E-36	1.52E-34
Hden_2684	hypothetical protein	2.33	1.22	1.06E-16	2.70E-15
Hden_2786	hypothetical protein	4.11	2.04	4.69E-28	1.69E-26
Hden_2822	efflux RND transporter periplasmic adaptor subunit	3.69	1.88	2.01E-03	9.02E-03
Hden_2931	potassium-transporting ATPase subunit KdpA	3.76	1.91	2.11E-06	2.03E-05
Hden_2933	potassium-transporting ATPase subunit KdpC	2.10	1.07	3.98E-02	1.05E-01
Hden_2938	formylglycine-generating enzyme family protein	2.14	1.09	9.57E-04	4.77E-03
Hden_2956	hypothetical protein	2.79	1.48	1.57E-02	5.02E-02
Hden_2958	glycoside hydrolase family 108 protein	2.06	1.04	2.02E-02	6.14E-02
Hden_2965	hypothetical protein	2.26	1.18	7.04E-02	1.64E-01
Hden_3023	hypothetical protein	3.14	1.65	4.24E-03	1.69E-02
Hden_3040	hypothetical protein	2.30	1.20	1.04E-11	1.99E-10
Hden_3139	hypothetical protein	3.35	1.74	1.30E-70	9.15E-69
Hden_3465	hypothetical protein	2.18	1.12	8.00E-12	1.54E-10
Hden_R0052		2.48	1.31	6.69E-05	4.66E-04
none_0000	DUF1488 family protein	3.48	1.80	3.67E-25	1.24E-23

Supplementary Table 3. Strains, primers and plasmids

Strains primers or plasmids	Relevant genotype, description or sequence	Reference or source
**Strains**		
Escherichia coli 10-beta	*Δ(ara-leu) 7697 araD139 fhuA ΔlacX74 galK16 galE15 e14- φ80dlacZΔM15 recA1 relA1 endA1 nupG rpsL* (Str^R) *rph spoT1 Δ(mrr-hsdRMS-mcrBC)*	New England Biolabs
E. coli BL21 DE3	*B dcm ompT hsdS*($r_B^- m_B^-$) *gal*	Novagen
Hyphomicrobium denitrificans ΔtsdA	Sm^r, in-frame deletion of *tsdA* in *H. denitrificans* Sm200	[6]
H. denitrificans ΔtsdA ΔshdrR	Sm^R, in-frame deletion of *shdrR* (Hden_0682) in *H. denitrificans ΔtsdA*	[7]
H. denitrificans ΔtsdA ΔsoxR	Sm^R, deletion of *soxR* (Hden_0700) in *H. denitrificans ΔtsdA*	[8]
H. denitrificans ΔtsdA ΔsoxT1B	Sm^R, in-frame deletion of *soxT1B* (Hden_0699) in *H. denitrificans ΔtsdA*	This work
Hyphomicrobium denitrificans ΔtsdA soxT1Bcomp	Sm^R, cis complementation of *H. denitrificans ΔtsdA ΔsoxT1B* with *soxT1B*	This work
H. denitrificans ΔtsdA soxT1B-Cys^{24}*Ser*	Exchange of SoxT1B-Cys^{24} to Ser in *H. denitrificans ΔtsdA*	This work
H. denitrificans ΔtsdA soxT1B-Cys^{98}*Ser*	Exchange of SoxT1B-Cys^{98} to Ser in *H. denitrificans ΔtsdA*	This work
H. denitrificans ΔtsdA soxT1B-Cys^{304}*Ser*	Exchange of SoxT1B-Cys^{304} to Ser in *H. denitrificans ΔtsdA*	This work
H. denitrificans ΔtsdA ΔsoxT1B ΔsoxR	Sm^R, simultaneous deletion of *soxR* (Hden_0700) and *soxT1B* (Hden_0699) in *H. denitrificans ΔtsdA*	This work
H. denitrificans ΔtsdA ΔsoxT1B ΔshdrR	Sm^R, simultaneous deletion of *shdrR* (Hden_0682) and *soxT1B* (Hden_0699) in *H. denitrificans ΔtsdA*	This work
H. denitrificans ΔtsdA ΔsoxT1A	Sm^R, in-frame deletion of *soxT1A* (Hden_0681) in *H. denitrificans ΔtsdA*	This work
H. denitrificans ΔtsdA ΔsoxT1A ΔsoxR	Sm^R, deletion of *soxR* in *H. denitrificans ΔtsdA ΔsoxT1A*	This work
H. denitrificans ΔtsdA ΔsoxT1A ΔshdrR	Sm^R, deletion of *shdrR* in *H. denitrificans ΔtsdA ΔsoxT1A*	This work
**Primers**		
SoxT1B_Del_Up_Fw	AATA**TCTAGA**CGAGCGATCGCCATCGCGAG (XbaI)	This work
SoxT1B_Del_Up_Rev	CGCCCGCATGCCAATCAGCTGATCATCGGAATTCGCTCTCT	This work
SoxT1B_Del_Down_Fw	AGAGAGCGATTCCGATGATCAGCTGATTGGCATGCGGGCG	This work
SoxT1B_Del_Down_Rev	ATCT**CTGCAG**TTCGAACCTGGACGCCGCG (PstI)	This work
SoxT1A_del_Up_Fw	GTGG**TCTAGA**TGTTCAAGCTCCTCGACAAG (XbaI)	This work
SoxT1A _del_Up_Rev	GCCGCCTGTCGTTCTTAAATCCGCATTTCCCGCCCCGTCT	This work
SoxT1A_del_Down_Fw	AGACGGGGCGGGAAATGCGGATTTAAGAACGACAGGCGGC	This work
SoxT1A_del_Down_Rev	GTGG**GTCGAC**CCTTTCTGGTCCATCAATGC (SalI)	This work
SoxT1B _C24S_Up_Rev	GGCGCCGCCGCCCGCTACGGACATCTCTCCTCCATGGGAG	This work
SoxT1B_C24S_Down_Fw	CTCCCATGGAGGAGAGATGTCCGTAGCGGGCGGCGGCGC C	This work
SoxT1B C98S_Up_rev	GGCACATCCAGCTTCGGGTTGCTCGTGCGCCTC	This work
SoxT1B_C98S_Down_Fw	GAGGCGCACGAGCAACCCGAAGCTGGATGTGCC	This work
SoxT1B_C304S_Up_rev	AAGGGCTCCACCATCGGCCAAGGCATGAGCGCCGGC	This work
SoxT1B_C304S_Down_Fw	GCCGGCGCTCATGCCTTGGCCGATGGTGGAGCCCTT	This work
P1 fwd up hden_0700	TATA**CTGCAG**GATCAAGGACGTGGTGGCG (PstI)	[8]
P5 fwd down hden_soxR/soxT1B	CCAGGGATAGGAATGTCAGCTGATTGGCATGCGGGC	This work
P6 rev down hden_ soxR/soxT1B	TTGC**TCTAGA**TCCGGCGCGACGATCGATG (XbaI)	This work
P7 rev up hden_ soxR/soxT1B	GCCCGCATGCCAATCAGCTGACATTCCTATCCCTCGG	This work
rpoB-denitf	AGGACGTGTTCACCTCGATT	[9]
rpoB-denitr	CGGCTTCGTCAAGGTTCTTC	[9]
SoxT1A 0681_qPCR-Fr	CCCGAGTGATACGATTCGCA	[8]
SoxT1A 0681_qPCR-Rev	CTAAAATGCCGCCGGTGATG	[8]
sHdrA_qPCR-Fr	CCGATCACCATTCCGTTCGA	[8]
sHdrA_qPCR-Rev	CAATTGTTTCCGGGCCGATC	[8]
SoxXA_qPCR-Fr	CGGCGCTCATTACCTATCTC	[8]
SoxXA_qPCR-Rev	TCGGGGTGTCTTTTTCAGTC	[8]
SoxT1B (0699)_qPCR-Fr	GCCGCCGTCTCAGTAAATAA	[8]
SoxT1B (0699)_qPCR-Rev	AGCAGAAGACGGCAGATGAT	[8]

Plasmids

pHP45Ω-Tc	Ap^r, Tc^r	10
pk18*mobsacB*	Km^r, Mob^+, *sacB*, *oriV*, *oriT*, *lacZα*	11
pk18*mobsacB*-Tc	Km^r, Tc^r pHP45Ω-Tc tetracycline cassette inserted into pk18*mobsacB* using SmaI	7
pk18*mobsacB*ΔtsdATc	Km^r, Tc^r, 2.01 kb fragment implementing deletion of a 996 bp *tsdA* fragment in pK18*mobsacB* with additional tetracycline resistance	6
pk18*mobsacB*-Δs*oxT1A*	Km^r, 2.07 kb SOE PCR fragment implementing *in-frame* deletion of nucleotides encoding amino acids 3 to 361 of SoxT1A cloned into XbaI and SalI of pk18*mobsacB*	This work
pk18*mobsacB*-Δs*oxT1A*-Tc	Km^r, Tc^r, pk18*mobsacB*-Δ*soxT1A* with tetracycline resistance gene from pHP45Ω cloned into SmaI site	This work
pk18*mobsacB*-Δ*soxT1B*	Km^r, 2.07 kb SOE PCR fragment implementing *in-frame* deletion of nucleotides encoding amino acids 3 to 350 of SoxT1B cloned into XbaI and PstI of pk18*mobsacB*	This work
pk18*mobsacB*-Δ*soxT1B*-Tc	Km^r, Tc^r, pHP45Ω-Tc tetracycline cassette inserted into pk18*mobsacB*-Δ*soxT1B* using SmaI	This work
pk18*mobsacB-soxT1Bcomp-Tc*	Km^r, Tc^r, SOE PCR fragment implementing chromosomal integration of *soxT1B* cloned into pk18*mobsacB*-Tc using XbaI and PstI restriction sites	This work
pk18*mobsacB-soxT1B-C24S*-Tc	Km^r, Tc^r, SOE PCR fragment implementing chromosomal integration of *soxT1B* encoding a Cys^{24}Ser exchange cloned into pk18*mobsacB*-Tc using XbaI and PstI restriction sites	This work
pk18*mobsacB-soxT1B-C98S*-Tc	Km^r, Tc^r, SOE PCR fragment implementing chromosomal integration of *soxT1B* encoding a Cys^{98}Ser exchange cloned into pk18*mobsacB*-Tc using XbaI and PstI restriction sites	This work
pk18*mobsacB-soxT1B-C304S*-Tc	Km^r, Tc^r, SOE PCR fragment implementing chromosomal integration of *soxT1B* encoding a Cys^{304}Ser exchange cloned into pk18*mobsacB*-Tc using XbaI and PstI restriction sites	This work
pk18*mobsacB*_Tc_Δ*soxR* (Hden0700)	Km^r, Tc^r, 1.04 kb SOE PCR fragment implementing deletion of nucleotides 4 to 362 of *soxR* to cloned into pk18*mobsacB*-Tc using XbaI and PstI restriction sites	8
pk18*mobsacB*ΔshdR-Tc	Km^r, Tc^r, 2.2 kb BamHI/XbaI fragment of PCR-amplified genome region around *shdrR* with deletion of *shdrR* cloned into BamHI/XbaI of pk18*mobsacB*	7
pk18*mobsacB*_Tc_Δ*soxR/soxT1B*	Km^r, Tc^r, 1.04 kb SOE PCR fragment implementing deletion of nucleotides 4 to of SoxR to nucleotide 1050 of *soxT1B* cloned into pk18*mobsacB*-Tc using XbaI and PstI restriction sites	This work

References

1. Tanaka, Y. *et al.* Crystal structure of a YeeE/YedE family protein engaged in thiosulfate uptake. *Science Advances* **6**, eaba7637 (2020).
2. Jumper, J. *et al.* Highly accurate protein structure prediction with AlphaFold. *Nature* **596**, 583-589 (2021).
3. Pettersen, E.F. *et al.* UCSF Chimera--a visualization system for exploratory research and analysis. *J. Comput. Chem.* **25**, 1605-12 (2004).
4. Madeira, F. *et al.* The EMBL-EBI Job Dispatcher sequence analysis tools framework in 2024. *Nucleic Acids Res.*, gkae241 (2024).
5. Rother, D., Heinrich, H.J., Quentmeier, A., Bardischewsky, F. & Friedrich, C.G. Novel genes of the *sox* gene cluster, mutagenesis of the flavoprotein SoxF, and evidence for a general sulfur-oxidizing system in *Paracoccus pantotrophus* GB17. *J. Bacteriol.* **183**, 4499-4508 (2001).
6. Koch, T. & Dahl, C. A novel bacterial sulfur oxidation pathway provides a new link between the cycles of organic and inorganic sulfur compounds. *ISME J.* **12**, 2479-2491 (2018).
7. Li, J. *et al.* A metabolic puzzle: consumption of C_1 compounds and thiosulfate in *Hyphomicrobium denitrificans* X^T. *Biochim. Biophys. Acta Bioenerget.* **1864**, 148932 (2023).
8. Li, J. *et al.* In the Alphaproteobacterium *Hyphomicrobium denitrificans* SoxR serves as a sulfane sulfur-responsive repressor of sulfur oxidation. *Antioxidants* **12**, 1620 (2023).
9. Martineau, C., Mauffrey, F. & Villemur, R. Comparative analysis of denitrifying activities of *Hyphomicrobium nitrativorans*, *Hyphomicrobium denitrificans*, and *Hyphomicrobium zavarzinii*. *Appl. Environ. Microbiol.* **81**, 5003-5014 (2015).
10. Fellay, R., Frey, J. & Krisch, H.M. Interposon mutagenesis of soil and water bacteria: a family of DNA fragments designed for in vitro insertional mutagenesis of Gram-negative bacteria. *Gene* **52**, 147-154 (1987).
11. Schäfer, A. *et al.* Small mobilizable multi-purpose cloning vectors derived from the *Escherichia coli* plasmids pK18 and pK19: selection of defined deletions in the chromosome of *Corynebacterium glutamicum*. *Gene* **145**, 69-73 (1994).
