# Supplementary material for: YeeE-like bacterial SoxT proteins mediate sulfur import for oxidation and signal transduction
Source: Commun Biol. 2024 Nov 21;7:1548. doi: 10.1038/s42003-024-07270-7 (PMC11582611; doi:10.1038/s42003-024-07270-7)
Supplement: Supplementary file 3 — Description of Additional Supplementary File [file 42003_2024_7270_MOESM3_ESM.pdf]

Description of additional supplementary file

File name: Supplementary Data 1
Description: The source data behind Figure 2

File name: Supplementary Data2
Description: The source data behind the graphs in Figures 3, 4, 5, and 6 and Supplementary Figures 6, 7, and 8.
